# Safety and immunogenicity of a malignant catarrhal fever vaccine candidate expressing ovine gammaherpesvirus-2 glycoprotein B in cattle

**DOI:** 10.3389/fimmu.2026.1895085

**Published:** 2026-09-10

**Authors:** Daniela D. Moré, Naomi S. Taus, Katherine N. Baker, Maria K. Herndon, Reginaldo G. Bastos, Benjamin G. Dewals, Alain Vanderplasschen, Cristina W. Cunha

**Affiliations:** 1 Animal Disease Research Unit, Agricultural Research Service, United States Department of Agriculture, Pullman, WA, United States; 2 Department of Veterinary Microbiology and Pathology, Washington State University, Pullman, WA, United States; 3 Department of Infectious and Parasitic Diseases, University of Liège, Liège, Belgium

**Keywords:** cattle, cellular immunity, glycoprotein B, humoral immunity, malignant catarrhal fever, ovine gammaherpesvirus 2, recombinant vaccine

## Abstract

**Background:**

Infection of susceptible livestock species, including domestic cattle and American bison, with ovine gammaherpesvirus-2 (OvHV-2), a gammaherpesvirus in the genus Macavirus, can result in an often-fatal disease known as sheep-associated malignant catarrhal fever (SA-MCF). Prevention of SA-MCF via vaccination is a key goal to support livestock producers.

**Methods:**

We assessed the safety and immunogenicity of a viral-vectored vaccine candidate consisting of a non-pathogenic recombinant alcelaphine gammaherpesvirus-1 expressing the OvHV-2 glycoprotein B (rAlHV-1/OvHV-2gB). The vaccine, formulated with an oil-in-water adjuvant, was administered intramuscularly to cattle using a prime-plus-two-boosters regimen.

**Results:**

Immunized animals did not develop MCF following rAlHV-1/OvHV-2gB inoculation. The vaccine virus did not establish persistent infection, and no viral shedding was detected after any immunization. Minor and transient adverse reactions associated with the adjuvant use were observed in some animals. Immunization with rAlHV-1/OvHV-2gB significantly altered the proportions of monocytes, T- and B- cells and induced the production of IFN-γ, CCL4, and CXCL10 in blood. Following *in vitro* stimulation with rAlHV-1/OvHV-2gB, peripheral blood mononuclear cells from immunized animals exhibited proliferation of CD4^+^ and CD8^+^ cells, along with production of both pro- and anti-inflammatory cytokines and chemokines. OvHV-2 gB-specific antibodies were present in both blood and nasal secretions, with neutralizing antibodies detected only in blood.

**Conclusion:**

Despite the need for further optimization of vaccine formulation and delivery, the rAlHV-1/OvHV-2gB vaccine elicited robust immune responses, supporting further evaluation of its protective efficacy in vaccine-challenge trials.

## 1 Introduction

Malignant catarrhal fever (MCF) is a frequently fatal lymphoproliferative disease of some ungulates, including domestic cattle (*Bos taurus* and *Bos indicus*) and American bison (*Bison bison*), caused by a group of gammaherpesvirus in the genus *Macavirus*. Among the MCF viruses, ovine gammaherpervirus-2 (OvHV-2; species *Macavirus ovinegamma2*) and alcelaphine gammaherpesvirus-1 (AlHV-1; species *Macavirus alcelaphinegamma1*) are the most frequently associated with disease. Domestic sheep (*Ovis aries*), which typically do not develop MCF, can be persistently infected and serve as reservoirs of OvHV-2. Transmission of the virus from sheep to clinically susceptible species can result in sheep-associated MCF (SA-MCF). Clinical and pathological manifestations of the disease have been well characterized (1). Clinically, the disease varies somewhat between susceptible species and can include ocular and nasal discharge, fever, inappetence, lethargy, diarrhea, difficulty urinating and hematuria. Multiple organs are affected, and ulceration of mucosal surfaces is common. Lymphoid hyperplasia and vasculitis are key histological features of SA-MCF. While cattle are considered relatively resistant to SA-MCF and may develop a chronic form of the disease (2), highly susceptible species, such as bison, typically die within a few days following the onset of clinical signs.

There is currently neither treatment nor vaccine against SA-MCF. Livestock producers rely on management practices to limit virus transmission between sheep and clinically susceptible species, mostly by physically separating them. Besides imposing limitations and restrictions for producers, the method is often inefficient, in part because OvHV-2 is mainly spread via aerosols, and transmission can occur over distances greater than five kilometers (3). A vaccine that protects animals from SA-MCF would greatly benefit producers by allowing multiple species to co-graze or be kept in proximity. Therefore, there is a critical need for an effective MCF vaccine to support affected livestock industries.

Previous studies have demonstrated protection of cattle from the AlHV-1-induced disease, wildebeest-associated malignant catarrhal fever (WA-MCF), by vaccinating animals with a cell culture attenuated AlHV-1 (4–9). Collectively, these studies show that intramuscular prime-boost vaccination with the attenuated AlHV-1 in an oil-in-water adjuvant induces circulating and mucosal humoral responses, which correlate with protection of animals from experimentally or naturally acquired WA-MCF. These studies demonstrate that protection against MCF through vaccination is possible; however, this attenuated AlHV-1 vaccine is not expected to protect against SA-MCF, since neutralizing antibodies against AlHV-1 and OvHV-2 do not cross react (10).

Because there is no *in vitro* system to propagate OvHV-2, it is not possible to produce a cell culture–attenuated vaccine for SA-MCF. Consequently, we explored the use of viral vectors as an alternative to deliver OvHV-2 antigens of interest as a vaccine for SA-MCF. Two non-pathogenic recombinant viruses, bovine herpesvirus-4 (BoHV-4; species *Rhadinovirus bovinegamma4*) lacking the thymidine kinase gene (11) and AlHV-1 lacking ORF73 (AlHV-1null73) (12), have been engineered to express OvHV-2 glycoprotein B (gB) and evaluated as vaccines in a rabbit model of SA-MCF (13–15). Both recombinant viruses were able to infect rabbits and induce immune responses, with protection rates upon OvHV-2 challenge varying from 43% to 71% for the BoHV-4 and the AlHV-1-vectored vaccine candidates, respectively (13, 14). Given the promising efficacy results in the rabbit study using the recombinant AlHV-1null73 expressing OvHV-2 gB (rAlHV-1/OvHV-2gB), we elected to further evaluate this vaccine candidate in a natural host.

In this study, we report the first evaluation of the safety and immunogenicity of the live rAlHV-1/OvHV-2gB chimera in cattle. The vaccine candidate, formulated with an oil-in-water adjuvant and administered intramuscularly, demonstrated a favorable safety profile and induced significant immune responses in vaccinated animals. A separate pilot study also provided a preliminary evaluation of the rAlHV-1/OvHV-2gB vaccine formulated without adjuvant and delivered by alternative routes.

## 2 Materials and methods

### 2.1 Viruses and cell lines

The recombinant virus used as a vaccine vector, rAlHV-1/OvHV-2gB, consisted of the AlHV-1null73 viral genome as a backbone with its gB-encoding gene replaced by the OvHV-2 homolog, as previously described (12, 16). For vaccine preparation, the virus was propagated in immortalized fetal mouflon sheep kidney cells (FMSKhtert.1) (17) using rAlHV-1/OvHV-2gB viral stocks previously prepared in our laboratory (16). Briefly, the virus was cultured in complete Dulbecco’s Modified Eagle Medium (cDMEM) containing glutamine, 10% fetal bovine serum (FBS), 100 U/mL penicillin, 100 µg/mL streptomycin and 1 µg/mL amphotericin B, and incubated at 37 °C and 5% CO_2_ until approximately 80% cytopathic effect was observed (6 to 10 days post-infection). Viral stocks were harvested by freezing and thawing cultures three times, clarified by centrifugation (3000 x g, 6 °C, 30 min) and stored at −80 °C until use. Virus preparations were quantified by a limiting dilution assay, and viral titers expressed as TCID_50_/mL.

Viability of the vaccine vector when mixed with the adjuvant used in this study was tested by incubating rAlHV-1/OvHV-2gB (10^4.7^ TCID_50_/mL) with or without 20% Emulsigen^®^ (Phibro Animal Health Corporation) at 20 °C for 1 h. Following incubation, virus preparations were titrated by limiting dilution assay.

BoHV-4/OvHV-2gB (15), used in the virus neutralization assay, was also propagated on FMSKhtert.1 cells as described above. Virus-containing supernatants were collected when cultures reached approximately 90% cytopathic effect (8–10 days post-infection) and clarified by centrifugation at 1000 x g for 15 minutes. Madin-Darby bovine kidney cells (MDBK, American Type Culture Collection), used for virus neutralization assays, were maintained in cDMEM at 37 °C and 5% CO_2_.

### 2.2 Animals and immunizations

All animal work and care were conducted under an animal use protocol approved by the Washington State University Institutional Animal Care and Use Committee (Animal Subject Approval Form # 7080). Eight (four males and four females) 4–5-month-old Jersey-Charolais cross calves (*Bos taurus*) were immunized three times at 14-day intervals by intramuscular injection (10 mL) in the neck containing either rAlHV-1/OvHV-2gB (8 x 10_5_ TCID_50_) in 20% Emulsigen® (Vac; n = 4) or Emulsigen® only (20%) in cell culture medium (DMEM) (Mock; n = 4). The virus used in all immunizations was from the same batch production. During the third immunization, a modification in the experimental design was necessary due to the development of anaphylactic-like reactions in the first two calves in the Mock group that were injected with DMEM/Emulsigen^®^. Following this, to protect animal welfare, the two remaining animals in the Mock group and all the animals in the Vac group were injected with DMEM or virus, respectively, without adjuvant. Animals were monitored daily for general body condition, behavior, and food and water intake. After each immunization, they were more closely evaluated for development of fever, injection site reactions, and lymph node enlargement.

We also performed a pilot experiment where animals were immunized with the rAlHV-1/OvHV-2gB formulated without adjuvant and administrated either intramuscularly or intranasally. Methods and results for this trial are described as **Supplementary Material** (Pilot Experiment and **Supplementary Figures 1, 2**).

### 2.3 Sample collection and processing

Blood and nasal secretions were obtained throughout the experiment (**Supplementary Figure 3**) from -1 to 84 days post-prime (DPI). Blood was collected from the jugular vein using a vacutainer system into ethylenediaminetetraacetic acid (EDTA) or acid citrate dextrose (ACD) tubes. Samples were immediately processed to obtain plasma and buffy coats by centrifugation of EDTA-blood and peripheral blood mononuclear cells (PBMCs) from ACD-blood. PBMCs were isolated using gradient centrifugation of equal volumes of diluted buffy coats and Histopaque-1077 (Sigma, Darmstadt, Germany) at 900 x *g* for 30 minutes at 20 °C without brake. PBMCs were collected, washed, and incubated for one minute in Lysis Buffer (Qiagen, Germantown, USA) to remove remaining red blood cells. Cells were resuspended in freezing media (FBS-10% DMSO) and stored in liquid nitrogen until use.

Nasal secretions were collected using foam swabs (Contec, Thermo Fisher Scientific, Waltham, USA) that were placed in each nostril (one/nostril) for 30 sec and then stirred in one mL of phosphate buffered saline (PBS, Thermo Fisher Scientific, Waltham, USA). The secretion fluid was clarified by centrifugation and both supernatant and cell pellet stored at -20 °C until used for analysis.

### 2.4 Polymerase chain reaction

Persistence of the virus vector in immunized animals was monitored using qPCR. DNA was extracted from buffy coats and nasal secretion pellets using the QIAamp kit (Qiagen, Germantown, USA). Total DNA samples were quantified by fluorometry (Quant-iT ds DNA broad range assay, Thermo Fisher Scientific, Waltham, USA) using a Qubit fluorometer (Thermo Fisher Scientific, Waltham, USA) and tested by a qPCR targeting AlHV-1 ORF3, as previously described (18).

### 2.5 Complete blood count (CBC)

For an initial assessment of leukocyte populations and overall animal health monitoring, CBCs were performed daily for four days following each immunization, then every two to three days using a ProCyte Dx^®^ hematology analyzer (IDEXX Inc., Westbrook, USA). Cell count ranges for healthy cattle followed the reference values available in the IDEXX VetLab System.

### 2.6 Immunophenotyping analysis

Flow cytometry was used to characterize PBMC sub-populations based on the expression of surface markers, tested alone or in combination. Cells were examined at 3, 6, 17, 21, 31 and 34 DPI (three and six days post each immunization). Cells collected at 29 DPI were also phenotyped following stimulation *in vitro*. The following cellular markers were analyzed, and their corresponding monoclonal antibody clones used in this study were as follows: CD3 (MM1A), CD4 (CC8), CD8 (CC63), CD14 (CAM36A), CD16 (KD1), CD335 (EC1.1), γδ-TCR (GB21A), and IgM (BAQ155A). Detailed information on antibodies and dilutions used to detect the markers are presented in **Supplementary Table 1**. PBMCs were stained in 96-well plates and acquired (20,000 events) using a Cytoflex**^®^** Instrument (Beckman Coulter, Brea, USA). After acquisition, data were analyzed using FCS Express**^®^** software v.6 (*De Novo* Software, Pasadena, USA). The percentage of positive cells was obtained through a standard gating strategy, consisting of first gating singlets and then total PBMCs based on their size and granularity.

### 2.7 Cell stimulation and proliferation assay

Cell proliferation was assessed by culturing PBMCs in the presence of 5-ethynyl-2´-deoxyuridine (EdU). Incorporation of EdU into newly synthesized DNA was detected by flow cytometry using the Pacific Blue–conjugated Click-iT Plus EdU kit (Thermo Fisher Scientific, Waltham, MA, USA), following the manufacturer’s instructions. The protocol was modified by scaling down the procedure for use in 96-well plates. Briefly, PBMCs collected from Vac and Mock animals at 29 DPI were seeded (5 × 10^5^ cells per well) into 96-well plates in the presence of 10 µM EdU. Cultures were incubated under three stimulation conditions: medium only (negative control), concanavalin A (50 µg/mL; positive control), or rAlHV-1/OvHV-2gB (10^4^ TCID_50_/mL). For all conditions, cell viability was assessed using eF780-conjugated Fixable Viability Dye^®^ (Thermo Fisher Scientific, Waltham, MA, USA) according to the manufacturer’s instructions. After 72 h in culture, EdU incorporation and expression of surface markers in proliferating cells were assessed by flow cytometry as described above. For analysis, specific cell populations were selected based on membrane markers, and the percentage of EdU^+^ cells was then quantified within each subset. Results from both total PBMCs and specific cell subsets are presented as proliferation index, calculated by normalizing the percentage of proliferating (EdU^+^) cells following stimulation with rAlHV-1/OvHV-2gB to the percentage of proliferating cells in matched non-stimulated controls, which were set to 100%.

### 2.8 Cytokine and chemokine quantification

Production of cytokines and chemokines in plasma samples and supernatants of stimulated PBMCs was measured using a multiplex assay, MILLIPLEX^®^ Bovine Cytokine/Chemokine Panel, for detection of IFN-γ, IL-1α, IL-1β, IL-4, IL-6, CXCL8 (IL-8), IL-10, IL-17A, IL-36RA, CXCL10 (IP-10), CCL2 (MCP-1), CCL3 (MIP-1α), CCL4 (MIP-1β), TNF-α, and VEGF-A (Millipore-Sigma, Darmstadt, Germany), according to the manufacturer’s instructions. Reactions were read on an LX-200™ instrument (Luminex, Austin, TX, USA).

For plasma samples, collected at multiple time points between 0 and 32 DPI, results are presented as pg/mL. For supernatants of cells obtained 24 hours post-last immunization (29 DPI) and stimulated *in vitro* for 72 hours, data are shown as net concentration in pg/mL, which represents the difference between the cytokine concentration observed in virus-stimulated cells and matched non-stimulated cells.

### 2.9 OvHV-2 gB enzyme-linked immunosorbent assay

OvHV-2 gB specific IgG responses were measured by ELISA as previously described (13, 19). Briefly, protein extracts from 293T transfected cells expressing OvHV-2 gB and mock-transfected cells (no gB) were prepared and used at 1:400 dilution to coat 96-well ELISA plates. Plasma samples were diluted at 1:100 and tested on gB-coated wells. Due to high background, nasal secretions were tested at 1:40 on both gB-coated wells and mock-coated wells. A goat anti-bovine IgG (H+L)/HRP conjugate (Jackson ImmunoResearch Laboratories Inc, Bar Harbor, USA) diluted at 1:20,000 was used for detection. Results for plasma samples are reported as the ratio of sample optical density (OD) divided by the background OD (blank), whereas for nasal secretions the ratio of the sample OD (gB antigen OD subtracted from mock antigen OD) by the background OD was used.

### 2.10 Virus neutralization assay (VNA)

To assess the development of neutralizing antibodies in plasma and nasal secretions, VNA was performed using rBoHV-4/OvHV-2gB, as previously described except that MDBK cells were used (14). Briefly, heat inactivated (56 °C for 30 minutes) plasma or nasal secretions diluted at 1:8 were mixed with virus (10^2^ TCID_50_/well), in triplicates, and incubated at 37 °C for one hour. The mixtures were then added to MDBK cells (2 x 10^4^ cells/well) and incubated at 37 °C for 2 h. Virus mix was removed and cells overlayed with 1% carboxymethylcellulose in cDMEM and 1% FBS and incubated at 37 °C and 5% CO_2_ for eight days. Cells were fixed and stained, and plaques counted under light microscopy. Results are expressed as percent of inhibition calculated as: % inhibition = 100 – (X * 100/Max) where X is the average number of plaques counted in wells containing tested sample, and Max is the number of plaques counted in wells containing medium only. The assay cut off was calculated as 56% based on standard positive and negative samples.

### 2.11 Statistical analysis

Data for CBC, cytokine/chemokine production and antibody responses in blood were compared using 2-way repeated measures ANOVA, with Geisser-Greenhouse correction for lack of sphericity, followed by Tukey’s post-test to assess the differences between groups at each time point. For cases where values were missing, a mixed effects model was used. Proliferation indexes and cytokine/chemokine production in response to *in vitro* cell stimulation in the Vac and Mock groups were compared using t-test when both groups satisfied the normality assumption, or the non-parametric Mann–Whitney test when at least one group violated normality. Normality was evaluated using the Shapiro–Wilk test. For all analyses, p values less than 0.05 were considered significant. All statistical analyses and graphing were performed using GraphPad Prism Software version 10.6.1 for Windows (GraphPad Software, San Diego, USA).

## 3 Results

### 3.1 Immunization with live rAlHV-1/OvHV-2gB was safe for cattle

The vaccine candidate used in this study consists of a recombinant AlHV-1 as a vector expressing the OvHV-2 gB target antigen. Therefore, a key objective of the initial safety assessment was to confirm that the vaccine candidate did not induce MCF. We first showed that 20% Emulsigen^®^ did not affect viral viability *in vitro* (**Supplementary Figure 4**), confirming that the rAlHV-1/OvHV-2gB remained infectious after formulation. Despite retaining infectivity, the vaccine caused no signs of AlHV-1-induced MCF in any immunized animal, supporting the conclusion that the vaccine virus is attenuated and safe for use in cattle under the conditions of this study.

Other potential adverse effects of immunization were assessed based on local and systemic reactions. As shown in **Table 1**, animals in both groups exhibited transient injection-site swelling following the prime and/or first booster immunizations, and one animal in each group showed prescapular lymph node enlargement after the first booster. Some animals also developed fever 1–2 days after the prime and first booster immunizations; however, fever 7–8 days after the prime immunization was observed only in the Vac group (**Table 1**; **Supplementary Table 2**). Importantly, two animals in the Mock group exhibited transient anaphylactic-like reactions immediately after the injection of the second DMEM/Emulsigen^®^ booster, becoming ataxic with increased respiratory rates and collapsed to the ground, but they recovered without treatment within five minutes. No further adverse effects were observed in these two animals or in the remaining two calves in the Mock group and all four animals in the Vac group that received the second booster without the adjuvant.

**Table 1:** Immunization regimen and side effects observed in individual animals in the vaccinated (Vac) and mock groups.

Group	Cattle ID	Immunization [DPI]	Side effects [DPI]			
			Local		Systemic	
			Injection site	LN	Fever (°C)	Other
Vac	V1	Virus + adjuvant [0, 14] Virus [28]	–	+ [15,16]	40.2 [7]	–
	V2		+ [15]	–	40.1 [7], 39.4 [15]	–
	V3		+ [15]		39.8 [2], 39.7 [3], 39.5 [8], 39.7 [15]	–
	V4		+ [3,4,7,8,15]	–	40.3 [8]	–
Mock	M1	Medium + adjuvant [0, 14] Medium [28]	–	–	39.4 [2]	–
	M2		+ [1,15]	–	39.4 [1]	–
	M3	Medium + adjuvant [0, 14, 28]	–	–	–	+ [28]
	M4		+ [1,2,15,16]	+ [16,17]	–	+ [28]

Virus, rAlHV-1/OvHV-2gB; Adjuvant, Emulsigen^®^; Medium, DMEM.

DPI, days post-prime immunization. Immunizations were performed at 0, 14 and 28 DPI.

Injection Site: -, normal; +, swelling/nodules; ++, ulcerations/abscesses.

LN, prescapular lymph node: -, normal; +, enlarged.

Fever: ≥39.4°C.

Other: -, none; +, transient anaphylactic-like reaction.

To evaluate safety in terms of viral persistence and shedding, animals in the Vac group were tested for the presence of AlHV-1 DNA in blood and nasal secretions every two to three days from 0 to 38 DPI and no viral DNA was detected in any of the samples at any time point tested.

### 3.2 rAlHV-1/OvHV-2gB immunization induced peripheral blood leukocyte modulation and cytokine production

**Figure 1:**
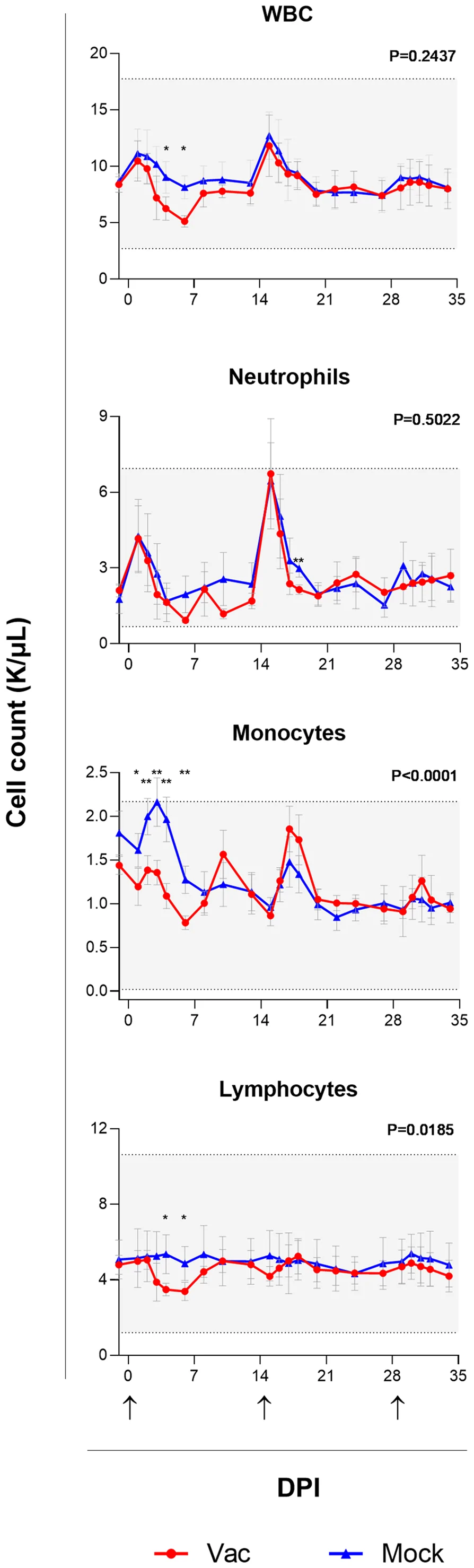
Modulation of peripheral blood cells following immunization. Calves were immunized intramuscularly with rAlHV-1/OvHV-2gB (Vac, n = 4, red circle) or with a mock formulation (Mock, n = 4, blue triangle) at days 0 and 14 (vaccine virus or medium plus Emulsigen^®^) and at day 28 (virus or medium only), as indicated by arrows. DPI stands for days post-prime immunization. Total white blood cells (WBC) counts and differential counts for neutrophils, monocytes, and lymphocytes were quantified throughout the experiment using a ProCyte Dx^®^ hematology analyzer. Gray areas indicate the reference ranges for each cell type in healthy cattle. Symbols represent group means, and error bars show standard deviation. Reported p-values correspond to the 2-way ANOVA interaction effect (time x group). Individual time points differences obtained by Tukey’s post-test comparisons between groups are indicated by asterisks: *p < 0.05; **p < 0.01.

Overall, immunization induced transient changes in total counts of white blood cells (WBC) and subpopulations in both Vac and Mock groups, with major alterations occurring after the prime and the first booster, which were delivered with adjuvant in both groups (**Figure 1**). Despite the fluctuations, all cell population values remained within normal reference ranges for healthy cattle (**Figure 1**, gray area). Significant differences in WBC counts between groups were observed only at 4 and 6 DPI (p = 0.0214 and p = 0.0045, respectively), when the Vac group exhibited lower cell levels than the Mock group. Neutrophil modulation was similar between groups (p = 0.5022), with counts peaking after the prime and first booster immunizations. Monocyte counts differed significantly between groups over time (p < 0.0001). This difference was primarily driven by changes during the first days following the prime immunization, when counts peaked exclusively in the Mock group before declining in both groups. Following the first booster immunization, both groups showed a transient increase in monocyte counts. Lymphocyte counts also differed significantly between groups over time (p < 0.0185), and at 4 and 6 DPI (p = 0.0311 and p = 0.0385, respectively) with lower values in the Vac group.

**Figure 2:**
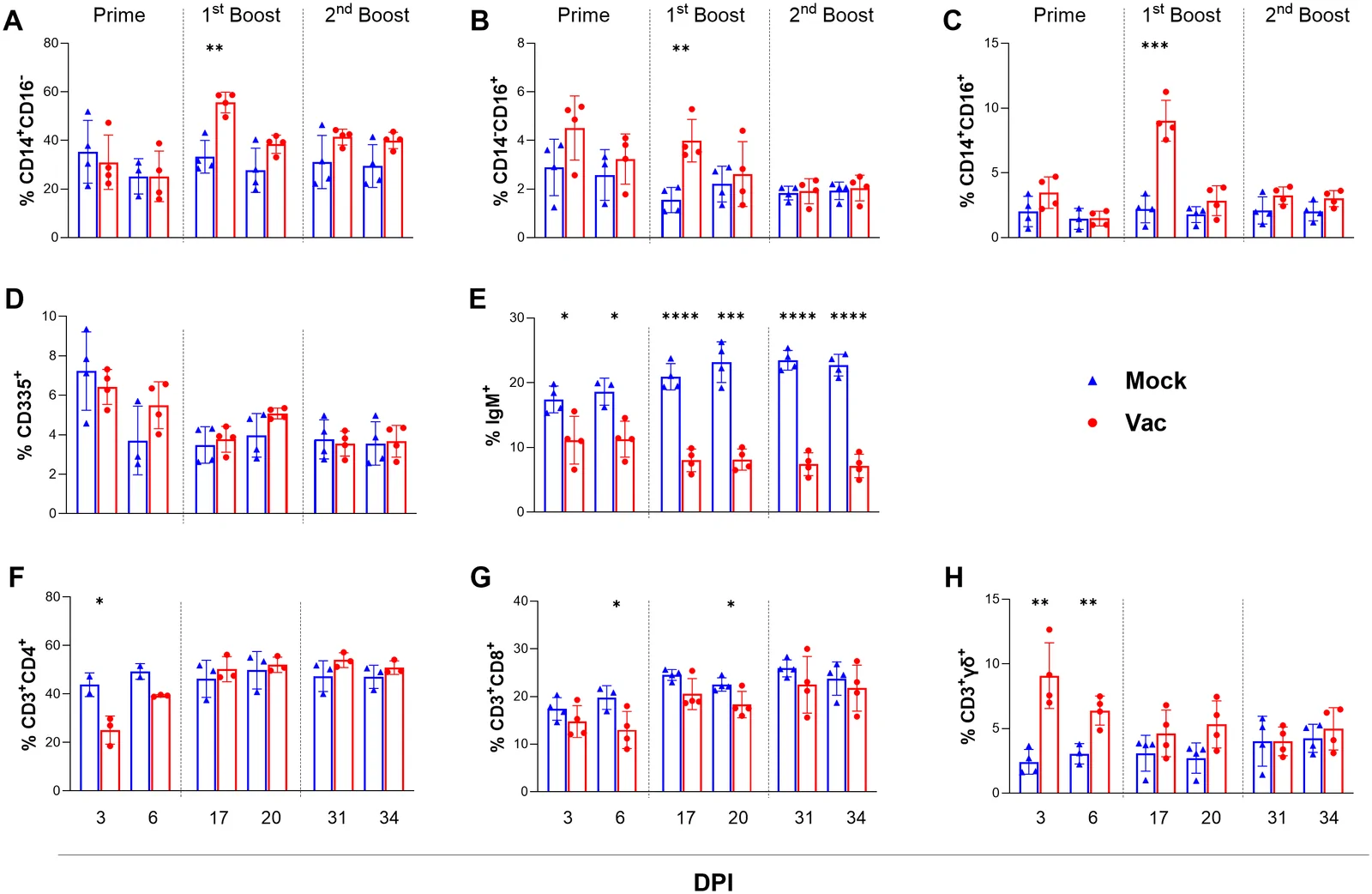
Immunophenotype of peripheral blood mononuclear cells (PBMCs) following rAlHV-1/OvHV-2gB or mock immunizations. The percentages of circulating monocyte and lymphocyte subpopulations were quantified by flow cytometry at 3 and 6 days post each immunization. DPI stands for days post-prime immunization. **(A)** CD14^+^CD16^-^ (classical), **(B)** CD14^-^CD16^+^ (non-classical) and **(C)** CD14^+^CD16^+^ (intermediate) monocyte subsets; **(D)** CD335^+^ cells; and **(E)** IgM^+^ B cells were quantified in PMBCs. **(F–H)** CD3^+^ cells were gated among total PBMCs, and the membrane co-expression of **(F)** CD4, **(G)** CD8, and **(H)** γδ-TCR were assessed. Symbols represent individual animals; bars indicate group means with error bars showing standard deviation. Statistical significance between groups (t-test) is indicated by asterisks: *p < 0.05; **p < 0.01; ***p < 0.001; ****p < 0.0001.

To better define the cellular modulation detected in the CBC analysis, PBMCs were further immunophenotyped three and six days after each immunization (3, 6, 17, 20, 31, and 34 DPI; **Figure 2**). Classical (CD14^-^CD16^+^), intermediate (CD14^+^CD16^+^), and non-classical (CD14^-^CD16^+^) monocyte subsets increased significantly (p < 0.01) in the Vac group compared to the Mock group three days after the first booster, 17 DPI (**Figures 2A–C**). No differences were observed in the percentage of CD335^+^ cells at any time point (**Figure 2D**). In contrast, the percentages of IgM^+^ cells (immature B cells) were significantly lower in the Vac group than in the Mock group following both the prime and booster immunizations (**Figure 2E**). T-cell subsets also showed group-dependent variation; CD3^+^CD4^+^ double-positive cells were reduced in the Vac group at 3 DPI (**Figure 2F**), while CD3^+^CD8^+^ cells were lower at 6 DPI and 20 DPI, corresponding to six days after the prime and first booster (**Figure 2G**). Conversely, CD3^+^γδ-TCR^+^ cell percentages were higher in the Vac group at 3 and 6 DPI compared to controls (**Figure 2H**).

**Figure 3:**
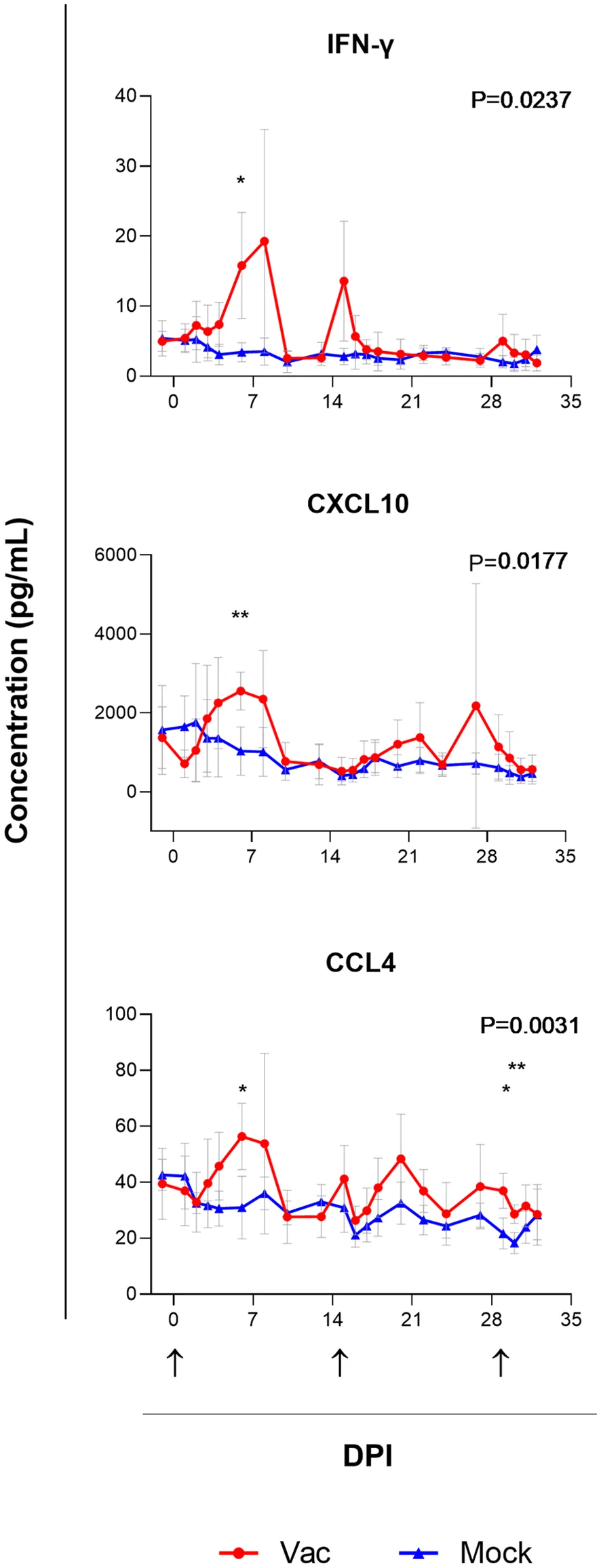
Immunization-induced modulation of cytokines and chemokines in plasma. Calves were immunized intramuscularly with rAlHV-1/OvHV-2gB (Vac, n = 4, red circle) or with a mock formulation (Mock, n = 4, blue triangle) at days 0 and 14 (vaccine virus or media plus Emulsigen^®^) and at day 28 (virus or medium only), as indicated by arrows. Cytokines and chemokines were quantified at multiple time points after immunizations using a MILLIPLEX^®^ multiplex assay; only those showing differential modulation between groups are presented. DPI stands for days post-prime immunization. Symbols represent the group means, and error bars indicate standard deviation. P values for the overall over time differences are shown, with asterisks indicating the time points where differences between groups were statistically significant as tested by 2-way repeated measures ANOVA, followed by Tukey’s post-test. *p < 0.05; **p < 0.01.

The development of cellular immune responses elicited by immunization was evaluated by measuring the levels of 15 cytokines and chemokines in plasma samples collected at multiple time points between 0 and 32 DPI. IFN-γ, CXCL10 and CCL4 were modulated over time (p = 0.0237, p = 0.0177, and p = 0.0031, respectively), with all three analytes showing increased levels in the Vac group at 6 DPI (p < 0.05), while CCL4 levels were also elevated at 29 and 30 DPI (p < 0.05) (**Figure 3**). IFN-γ was the only cytokine that was also significantly different (p = 0.0361) when the interaction between time and groups was considered. No substantial changes in average plasma levels of IL-1α, IL-1β, IL-4, IL-6, IL-10, IL-17A, IL-36RA, TNF-α, CXCL8, CCL2, or CCL3 were observed.

Taken together, the cell count data, immunophenotyping results and cytokine/chemokine profiles in blood samples indicate that immunization with rAlHV-1/OvHV-2gB elicited cellular immunity, resulting in a broad modulation of leukocyte populations and cytokine/chemokine production.

### 3.3 PBMCs from immunized animals differentially responded to *in vitro* rAlHV-1/OvHV-2gB stimulation

**Figure 4:**
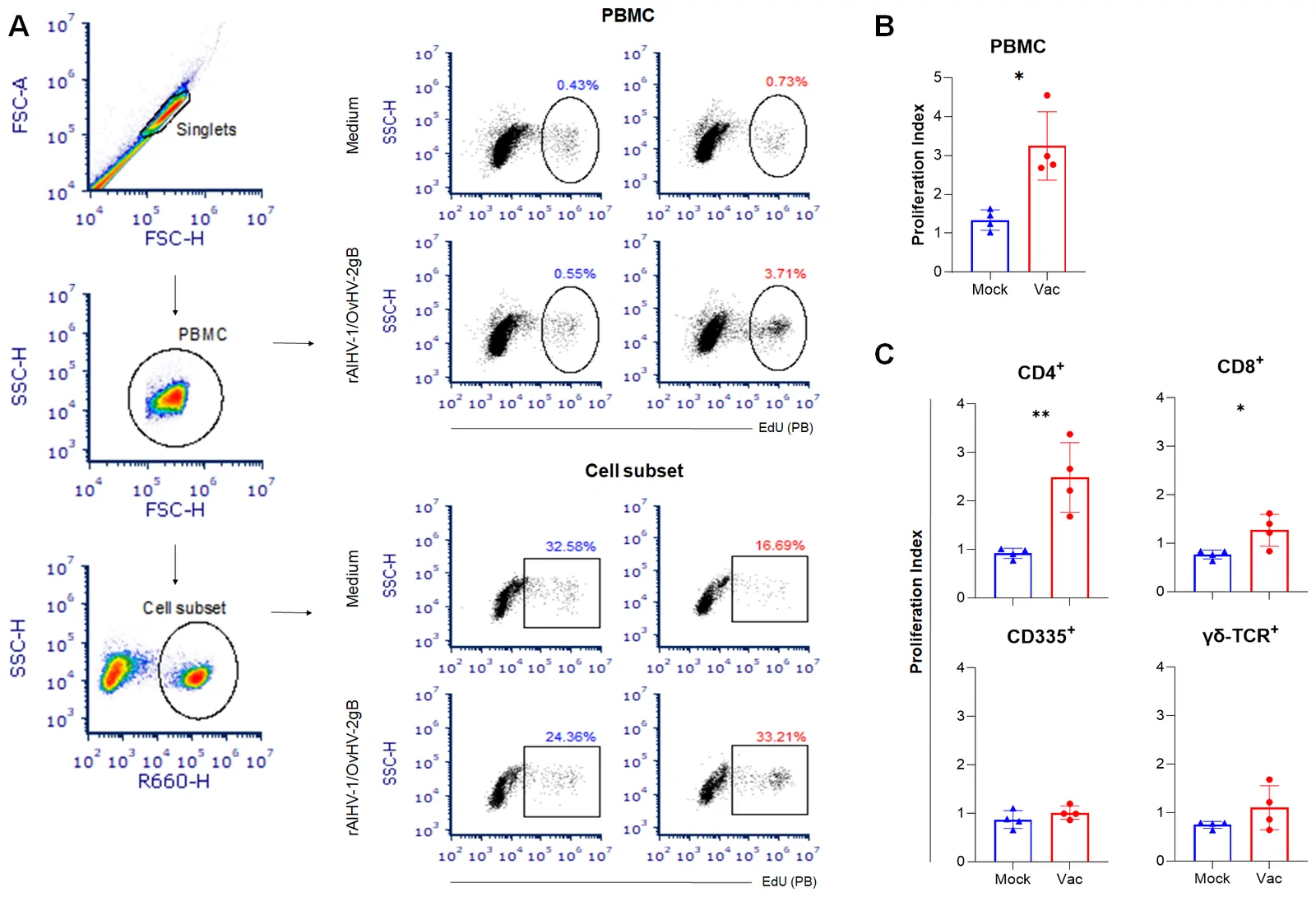
rAlHV-1/OvHV-2gB immunization resulted in differential cellular proliferation *in vitro*. Peripheral blood mononuclear cells (PBMCs) collected from Vac (n = 4; red circles) and Mock (n = 4; blue triangles) groups 24 hours post-last immunization were stimulated *in vitro* with rAlHV-1/OvHV-2gB. Proliferative responses were assessed by measuring EdU incorporation at 72 h post-stimulus using flow cytometry. **(A)** Representative gating strategy. A total of 20,000 PBMCs were selected among singlets based on their size (FSC) and granularity (SSC), and the percentage of EdU^+^ cells (PB: Pacific Blue) was determined for each group and condition. The total PBMC population was then used as the parent gate to identify cell subsets, based on the expression of CD4, CD8, CD335, and γδ-TCR markers. The percentage of EdU-positive cells was subsequently determined within each subpopulation. **(B)** The proliferation index in PBMCs was calculated as the ratio of the percentage of EdU^+^ cells in rAlHV-1/OvHV-2gB-stimulated cultures to that in unstimulated cultures for each animal. **(C)** Proliferation indexes for CD4^+^, CD8^+^, CD335^+^, and γδ-TCR^+^ cells were calculated as described above. Bars represent mean stimulation index, and error bars indicate standard deviation. Within each cell population, groups were compared using either t-test or the Mann-Whitney test and significant differences are represented by asterisk *p < 0.05; **p < 0.01.

Specific cellular responses induced by immunization were addressed through *in vitro* stimulation of PBMCs with rAlHV-1/OvHV-2gB (**Figure 4**), according to the gating strategy illustrated in **Figure 4A**. Total cell proliferation of PMBCs collected 24 hours after the last immunization (29 DPI) was higher in the Vac group than in the Mock (p = 0.0286), with mean proliferation indexes of 3.24 and 1.34, respectively (**Figure 4B**). The percentages of proliferating CD4^+^ and CD8^+^ cells were also significantly higher in the Vac compared to the Mock group, with proliferation indexes of 2.48 and 0.92 in CD4^+^EdU^+^ (p = 0.005) and 1.27 and 0.77 in CD8^+^EdU^+^ (p = 0.0265) for the Vac and Mock groups, respectively (**Figure 4C**). In contrast, no significant proliferation of CD335^+^ and γδ TCR^+^ cells was observed following stimulation with rAlHV-1/OvHV-2gB in either group. Importantly, cell viability, which would impact the assay, was not affected by exposure to the recombinant virus. These results suggest that immunization with rAlHV-1/OvHV-2gB primed CD4^+^ and CD8^+^ T cells that proliferate upon *in vitro* re-exposure to the vaccine virus.

**Figure 5:**
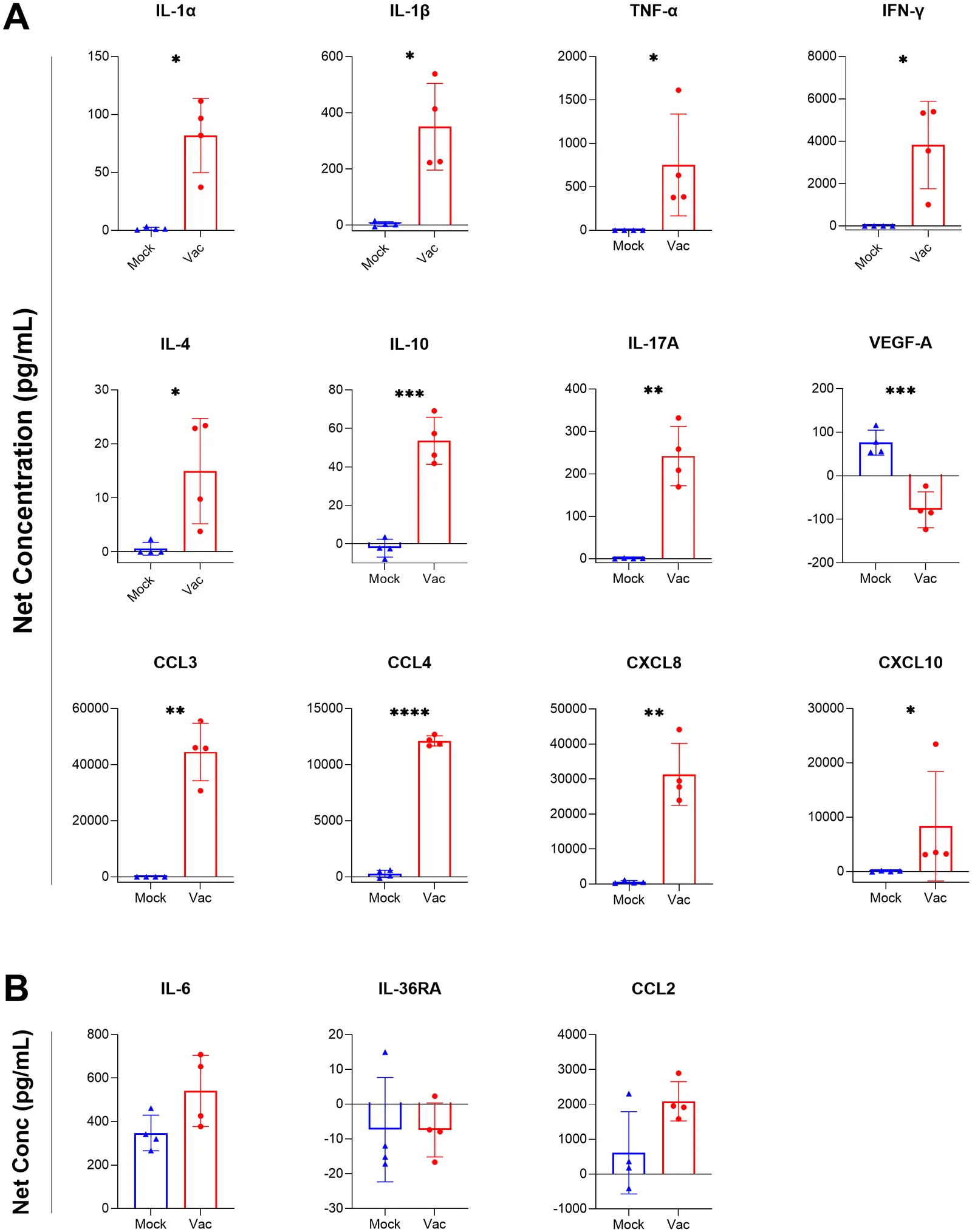
rAlHV-1/OvHV-2gB *in vitro* stimulation induced cytokine and chemokine production in immunized animals. PBMCs from animals immunized with rAlHV-1/OvHV-2gB plus adjuvant (Vac, n = 4, red circle) or cell culture medium plus adjuvant (Mock, n = 4, blue triangle), collected 24 hours post-last immunization (29 DPI), were stimulated *in vitro* with rAlHV-1/OvHV-2gB. Cytokine and chemokine concentrations in the supernatants were quantified using a MILLIPLEX^®^ multiplex assay. Results are presented as net concentration (concentration in virus-stimulated cells minus concentration in non-stimulated matched cells). Bars represent the mean ratios for each group, and error bars indicate standard deviations. Group comparisons were performed using either a t-test or a Mann-Whitney test and significant differences in panel A are indicated by asterisks (*p < 0.05; **p < 0.01; ***p < 0.001; ****p < 0.0001). No significant differences were observed for the cytokines and chemokines shown in panel B.

We also quantified the cytokines and chemokines secreted in supernatants of PBMC exposed to rAlHV-1/OvHV-2gB *in vitro* (**Figure 5**; **Supplementary Table 3**). Overall, cells from animals in both groups responded to the stimulus; however, cells from immunized animals exhibited a distinct profile, characterized by increased production of both proinflammatory and regulatory cytokines, and chemokines. As illustrated in **Figure 5A**, following virus exposure, the average production of IL-1α, IL-1β, TNF-α, IFN-γ, IL-4, IL-10, IL-17A, CCL3, CCL4, CXCL8, and CXCL10 was significantly higher in culture supernatants from Vac animals compared to Mock, while VEGF-A levels were significantly lower in the Vac group. No differences between groups were observed in the production of IL-6, IL-36RA, and CCL2 (**Figure 5B**).

Altogether, these results indicate that immunization with rAlHV-1/OvHV-2gB elicited cellular immunity activation, evidenced as an increased proliferative response and cytokine and chemokine production in vaccinated animals upon *in vitro* re-exposure to the recombinant virus.

### 3.4 Humoral responses elicited by immunization with rAlHV-1/OvHV-2gB in cattle included OvHV-2 gB-specific IgG and neutralizing antibodies

**Figure 6:**
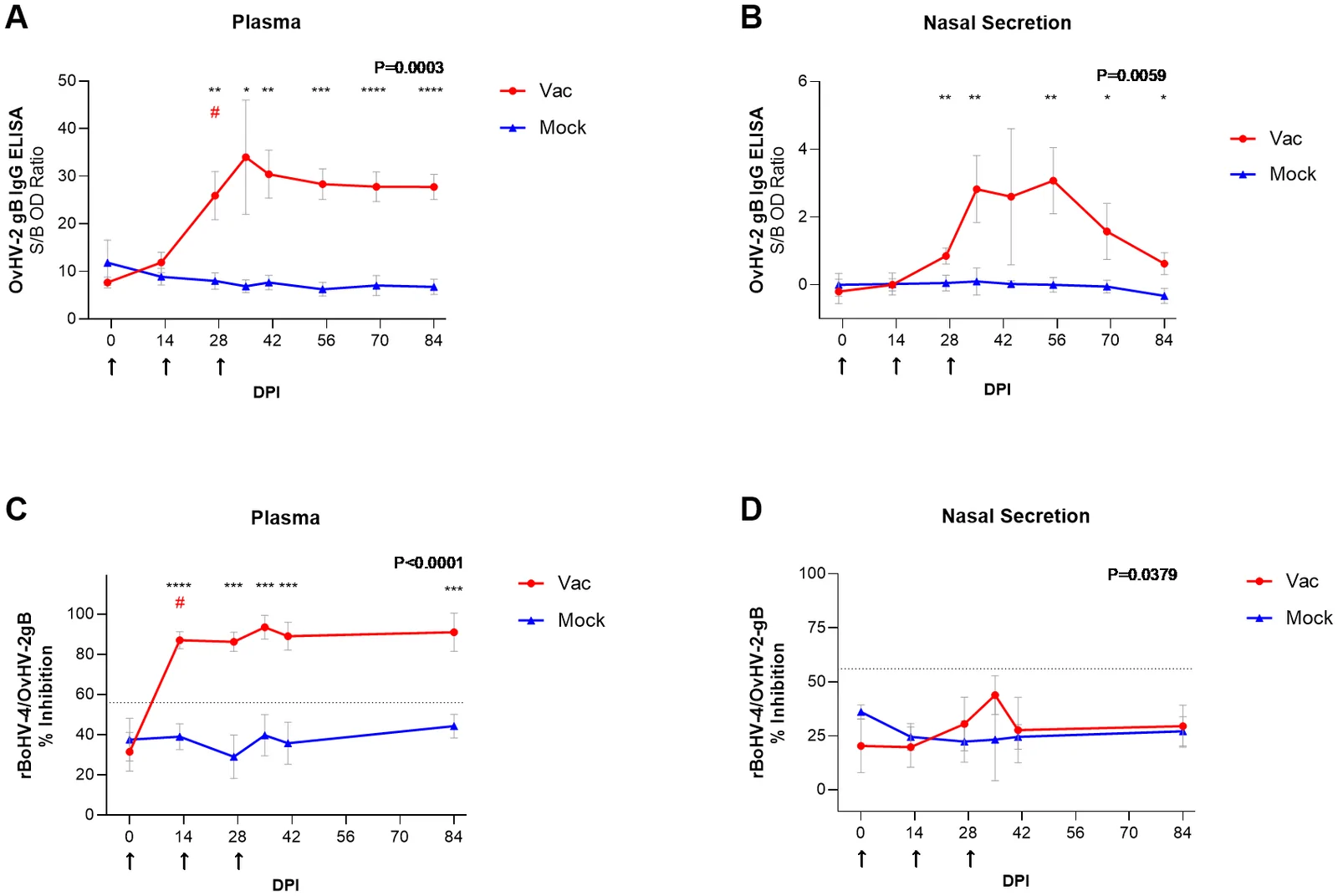
Humoral responses elicited by immunization with rAlHV-1/OvHV-2gB in cattle. Calves were immunized intramuscularly with rAlHV-1/OvHV-2gB (Vac, n = 4, red circle) or with a mock formulation (Mock, n = 4, blue triangle) at days 0 and 14 (vaccine virus or medium plus Emulsigen®) and at day 28 (virus or media only), as indicated by arrows. DPI stands for days post-prime immunization. Total IgG responses to OvHV-2-gB are shown in **(A)** plasma (1:100) and **(B)** nasal secretions (1:40). Results are mean Sample/Background OD450 ratio of OvHV-2 gB-specific IgG obtained by ELISA. Percentage of OvHV-2 gB-dependent inhibition of rBoHV-4/OvHV-2gB infection in a virus neutralization assay using **(C)** plasma and **(D)** nasal secretions, both diluted at 1:8; error bars are standard deviation; and dotted lines indicate the 56% inhibition cut off. Reported p-values correspond to the 2-way ANOVA interaction effect (time x group). Tukey’s post-test comparisons between groups at individual time points are indicated by asterisks (*p < 0.05; **p < 0.01; ***p < 0.001; ****p < 0.0001), whereas the hash symbol (#) denotes the first within-group significant difference from time zero, indicating seroconversion.

To determine whether immunization with rAlHV-1/OvHV-2gB stimulated the development of OvHV-2 gB-specific circulating and mucosal antibodies, plasma and nasal secretion samples were tested by ELISA. Detection of anti-OvHV-2 gB IgG in plasma of the Vac group started two weeks after the first booster at 27 DPI and high levels of OvHV-2 gB antibodies remained detectable through the end of the experiment (**Figure 6A**). Significant levels of IgG (p = 0.0031) were also observed in nasal secretions of animals in the Vac group following the first booster, at 27 DPI; however, the antibody level declined after 56 DPI (**Figure 6B**). Antibodies were not detected in either plasma or nasal secretions of animals in the Mock group at any time throughout the study. In the pilot experiment described in the **Supplementary Material**, intramuscular administration of rAlHV-1/OvHV-2gB without adjuvant elicited sustained OvHV-2 gB–specific IgG responses in the blood, whereas antibodies were not detected in nasal secretions (**Supplementary Figure 1**). Intranasal immunization in the absence of adjuvant did not induce detectable antibodies in any of these body compartments (**Supplementary Figure 1**).

The presence of OvHV-2 gB–specific neutralizing antibodies was evaluated in plasma and nasal secretions using an assay based on rBoHV-4/OvHV-2gB. As shown in **Figure 6C**, neutralizing activity was detected in plasma from Vac group animals after the first immunization (p = 0.0135) and remained significantly higher (86.2-93.5% inhibition) compared to the Mock group until the last samples tested at 84 DPI. Neutralizing activity was also detected in the plasma of vaccinated animals even in the absence of Emulsigen^®^ (**Supplementary Figure 2A**). In contrast, neutralizing antibody activity above the assay threshold was not observed in nasal secretions in any group (**Figure 6D**; **Supplementary Figure 2B**).

Collectively, these results show that rAlHV-1/OvHV-2gB when formulated with Emulsigen^®^ and delivered intramuscularly induced detectable humoral responses in the blood and respiratory tract, including circulating neutralizing activity. In contrast, removal of the adjuvant eliminated neutralizing activity in the blood, and intranasal delivery without adjuvant did not elicit measurable humoral responses.

## 4 Discussion

In this study, we demonstrated that rAlHV-1/OvHV-2gB did not cause significant adverse effects and elicited both cellular and humoral immune responses in cattle. We further discuss how these findings advance the evaluation of this candidate for a SA-MCF vaccine and outline future directions for comprehensive assessment, including efficacy studies in natural hosts.

The recombinant AlHV-1/OvHV-2gB did not persist in cattle following intramuscular immunization, did not cause MCF, and was not shed. These results confirm previous data that both the rAlHV-1/OvHV-2gB and its parental virus, rAlHV-1null73, are attenuated and unable to establish latency (12, 14, 20), which are important safety considerations, for both the animals and the environment. Although the absence of a persistent vaccine virus does not impede protective immunity, the use of adjuvants and booster immunizations is often required to achieve adequate immune stimulation (7, 21, 22). Following intramuscular immunization with rAlHV-1/OvHV-2gB in 20% Emulsigen^®^, minor and transient local reactions were observed in some animals. Notably, two calves in the Mock group experienced anaphylactic-like reactions immediately after the second booster injection. Because these animals received only the adjuvant in cell culture medium, the reactions were not attributable to the recombinant virus but prompted a reassessment of the vaccination protocol, resulting in removal of the adjuvant from the formulation given to the remaining animals to protect animal welfare. Similar reactions to Emulsigen^®^ have been reported previously in a trial using attenuated AlHV-1 formulated with 20% of the adjuvant, although in that study the adverse events occurred after the prime immunization (8). Although no cases of MCF were observed following immunization with the rAlHV-1/OvHV-2gB vaccine candidate, additional studies using larger number of animals are required to more thoroughly evaluate the safety profile of the vaccine formulation. Considering the potential risk of adverse effects following the use of adjuvants, proper choice of the product type, delivery, and regime must follow a comprehensive analysis to balance animal welfare and benefits of a proper immune response.

Although humoral responses to rAlHV-1/OvHV-2gB have been demonstrated in rabbits (14), cellular immunity to this chimera has not yet been characterized. Similarly, only limited descriptions of cellular immune responses in cattle vaccinated with cell-culture–attenuated AlHV-1 or the latency-deficient AlHV-1 backbone used in this study have been reported (20, 21). Therefore, expanding this knowledge is essential, as both innate and adaptive cellular immune responses may contribute to protection induced by vaccination. The current study advances this understanding by providing an initial characterization of cellular dynamics and cytokine and chemokine production along with the humoral response following vaccination with the rAlHV-1/OvHV-2gB chimera in cattle.

As illustrated in **Figure 2**, our data show that cattle vaccinated with rAlHV-1/OvHV-2gB exhibited a distinct peripheral blood cell profile compared to mock controls. Following immunization, vaccinated animals showed expansion of classical, intermediate, and nonclassical monocytes, as well as γδ T lymphocytes in peripheral blood, suggesting activation of innate immune functions and a role for these subsets in shaping antiviral adaptive immunity (23, 24). The contribution of γδ T cells to the control of gammaherpesvirus infections remains incompletely understood; although in ruminants they are known to produce proinflammatory and regulatory cytokines and have a role in memory responses, they have also been implicated in the pathogenesis of SA-MCF in bison (25). Therefore, the functional significance of the observed γδ T cell expansion following vaccination requires further investigation. In contrast, a reduction in the proportions of CD4^+^ and CD8^+^ T lymphocytes was detected in PBMCs of vaccinated animals relative to controls at some time points following the prime and first booster immunizations. Activation of CD4^+^ and CD8^+^ T lymphocytes is essential for controlling gammaherpesvirus infections by providing immune helper activity and direct antiviral effector functions, respectively; however, a lack of expansion of both CD4^+^ and CD8^+^ T lymphocytes populations was shown following rAlHV-1null73 infection in cattle, yet the animals were protected against challenge with wild-type AlHV-1 (20). Conversely, CD8^+^ T cells have also been implicated in disease pathogenesis (20, 26, 27). In the present study, the significant proportions of proliferating CD4^+^ and CD8^+^ cells observed in the Vac group following *in vitro* re-stimulation suggest the induction of effective recall immune responses. However, it is important to note that these responses were measured using the vaccine virus and therefore do not specifically reflect immunity directed against the OvHV-2 gB antigen. Confirmation of gB-specific responses would require stimulation with OvHV-2 or recombinant OvHV-2 gB, reagents that were not available for this study. Regarding the production of cytokines and chemokines, vaccination with rAlHV-1/OvHV-2gB led to a significant increase in plasma concentrations of IFN-γ, CXCL10, and CCL4 (**Figure 3**). While IFN-γ plays a well-established role in controlling herpesviruses (21, 28), the relevance of CXCL10 and CCL4 during herpesvirus infection remains to be determined. In addition, both proinflammatory (IFN-γ, IL-1α, and TNF-α) and regulatory cytokines (IL-4, IL-10, and IL-17A) were produced by cells from vaccinated animals following *in vitro* stimulation with rAlHV-1/OvHV-2gB (**Figure 5**). Together, these findings indicate that vaccination successfully primed immune cells and elicited recall responses that may contribute to protection. Notably, PBMCs from vaccinated animals produced significantly less VEGF-A than controls after *in vitro* stimulation with rAlHV-1/OvHV-2gB. Because gamma herpesviruses can induce VEGF-A to promote angiogenesis and viral infection (29), the significance of reduced VEGF-A production observed after vaccination warrants further studies as a potential mechanism of disease control. Finally, we observed a significant reduction in circulating immature B cells following immunization, suggesting their migration from peripheral blood to sites of infection and/or secondary lymphoid organs, where they may undergo activation and differentiation to produce antigen-specific antibodies that are critical for MCF protection upon challenge (5, 6, 21, 30).

Anti-OvHV-2 gB antibodies were detected in both plasma and nasal secretions of rAlHV-1/OvHV-2gB immunized animals; however, significant neutralizing activity was observed only in plasma (**Figure 6**). The limited or undetectable neutralizing responses in the respiratory tract are noteworthy, as a mucosal antibody barrier is expected to contribute substantially to protection against MCF (6). Previous work with the high-passage attenuated AlHV-1 has shown that vaccination induces strong systemic and mucosal antibody responses, but only neutralizing antibodies in nasal secretions correlate with protection (5, 6, 21). Moreover, IgA and IgG1 have been identified as the predominant isotypes in nasal secretions of protected animals vaccinated with attenuated AlHV-1 formulated with Freund’s adjuvant (5). In our study, we were unable to characterize antibody isotypes in nasal secretions; despite multiple attempts, reliable IgA measurements could not be obtained because of high assay background and the lack of standardized positive controls for proper optimization. Nevertheless, this remains a priority for future work, as defining the contribution of individual antibody isotypes within the pool of neutralizing antibodies may offer better correlates of protection than measuring total antibody levels. It is also important to note that the neutralizing antibody responses reported here reflect only inhibition associated with OvHV-2 gB. Responses targeting other viral antigens were not evaluated. Consequently, the potential role of antibodies unrelated to gB in protection against OvHV-2, or even AlHV-1, remains to be determined and should be addressed in future vaccine studies in which animals are challenged with either OvHV-2 or AlHV-1.

Our initial assessment of the role of adjuvant in intramuscular immunization with rAlHV-1/OvHV-2gB (pilot experiment described in **Supplementary Material**) showed more robust antibody responses when the vaccine candidate was administered with Emulsigen^®^ compared to virus alone (**Figure 6**, **Supplementary Figures 1**, **2**). This was evidenced by higher plasma neutralizing antibody titers and the presence of gB-specific antibodies in nasal secretions of animals in the vaccine-plus-adjuvant group, whereas no detectable antibodies were observed in animals receiving the virus alone. Emulsigen^®^ is an oil-in-water preparation widely used in studies of attenuated AlHV-1 vaccines, where it has consistently enhanced antibody responses associated with protection (6–9, 21). Although neutralizing antibodies were not detected in nasal secretions of any animals in our study, a recent comparison of cattle vaccinated with attenuated AlHV-1 with or without 20% Emulsigen^®^ reported that both formulations induced virus-specific antibodies, but the adjuvanted vaccine produced higher total and neutralizing antibody titers in plasma and nasal secretions (22). These findings suggest that inclusion of an adjuvant may enhance protective immunity; however, additional studies are needed to optimize the delivery route and adjuvant formulation for the rAlHV-1/OvHV-2gB vaccine candidate. Based on the safety and immunogenicity data reported here, we recommend that future evaluations limit Emulsigen^®^ to no more than two immunizations or reduce its concentration in the formulation to below 20%. These strategies would balance the immunogenic benefits of adjuvant use with the need to minimize adverse reactions, as reported here and in previous MCF vaccine studies (8). In addition, it would also be important to further evaluate strategies known to enhance upper respiratory tract immune responses, such as intranasal priming or mucosal adjuvants, while preserving the favorable cellular programming observed with intramuscular delivery.

In this study, evaluation of vaccine efficacy was not performed due to the lack of OvHV-2 at concentrations necessary to induce SA-MCF in cattle. Although protection with rAlHV-1/OvHV-2gB has been achieved in rabbits, used as a laboratory model, efficacy evaluation in natural hosts must be done in more susceptible hosts, such as bison. Because such studies are inherently more complex and costly, assessing safety and immunogenicity of the vaccine candidate in cattle was a crucial initial step to support advancing to efficacy trials in bison.

In conclusion, this initial safety assessment of the rAlHV-1/OvHV-2gB vaccine candidate in cattle found no indication of vaccine-associated MCF in the immunized animals. However, repeated administration of 20% Emulsigen® was associated with adverse effects, indicating that further optimization of vaccine delivery and adjuvant formulation is needed. Although the study included a limited number of animals, the findings provide encouraging preliminary safety data that support continued investigation of the rAlHV-1/OvHV-2gB as a vaccine candidate. Importantly, the vaccine, as tested, elicited robust cellular and humoral immune responses, warranting future vaccination-challenge studies aimed at determining efficacy and identifying correlates of protection for SA-MCF in natural hosts.

## Data Availability

The raw data supporting the conclusions of this article will be made available by the authors, without undue reservation.
