# Supplementary material for: Safety and immunogenicity of a malignant catarrhal fever vaccine candidate expressing ovine gammaherpesvirus-2 glycoprotein B in cattle
Source: Front Immunol. 2026 Sep 10;17:1895085. doi: 10.3389/fimmu.2026.1895085 (PMC13601324; doi:10.3389/fimmu.2026.1895085)
Supplement: Supplementary file 1 [file Supplementaryfile1.docx]

Supplementary Material

**Pilot Experiment:**

**Initial assessment of humoral responses induced by rAlHV-1/OvHV-2gB in the absence of adjuvant delivery by either intramuscular or intranasal routes.**

Methods: Eight (males) Holstein calves were immunized either intramuscularly (rAlHV-1/OvHV-2gB-IM_;_ n=4) or intranasally (rAlHV-1/OvHV-2gB-IN; n = 4) with 10 mL of rAlHV-1/OvHV-2gB (10^5^ TCID_50_/ml) without adjuvant every two weeks, for a total of three immunizations (at days 0, 14 and 28). For intranasal immunization, an atomization device (MADgic Laryngo-Tracheal Mucosal, Teleflex, Inc.) was used to deliver the vaccine into the nostrils. OvHV-2 gB-specific antibody responses, and neutralizing antibody activity were the only parameters assessed in these animals, which were performed as described in the Material and Methods section. For assessment of OvHV-2 gB specific antibody responses, plasma and nasal secretions were tested at 1:100 and 1:40 dilutions, respectively. To evaluate antibody levels among groups, selected samples were also serially diluted (1:8 to 1:256) and antibody titers considered as the reciprocal of the highest dilution that resulted in a percentage of inhibition higher than the assay cut off (56% inhibition).

Results: OvHV‑2gB IgG responses in plasma and nasal secretions for both rAlHV-1/OvHV-2gB-IM and -IN groups are shown in Supplementary Figure 3. Even without adjuvant, intramuscular immunization with rAlHV‑1/OvHV‑2gB induced OvHV‑2gB‑specific IgG responses in plasma. Antibody levels were significantly higher than pre‑immunization levels by one week after the first booster (21 DPI) and remained elevated until the last sampling at 35 DPI. In contrast, no OvHV‑2gB‑specific antibodies were detected in nasal secretions of these animals at any time point. Conversely, intranasal immunization with rAlHV‑1/OvHV‑2gB did not elicit detectable antigen‑specific antibodies in either plasma or nasal secretions, as antibody levels in both compartments remained comparable to pre‑immunization levels.

Neutralizing antibody activity in animals immunized without adjuvant was evaluated in plasma and nasal secretions at 0 and 35 DPI (Supplementary Figure 4). As illustrated in Supplementary Figure 4A, plasma from animals in the rAlHV‑1/OvHV‑2gB‑IM group exhibited an average of 94.2% inhibition at 35 DPI, significantly higher (p < 0.0001) than their pre‑immunization levels (average 29.7%). In the rAlHV‑1/OvHV‑2gB‑IN group, one animal showed neutralizing activity in plasma at 35 DPI (81% inhibition); however, the group average remained comparable to pre‑immunization values and below the positivity cutoff. Importantly, because rAlHV‑1/OvHV‑2gB does not establish persistent infection and animals immunized via the intranasal route did not develop antigen‑specific antibodies (Supplementary Figures 3 and 4B), we were unable to confirm infection in the rAlHV‑1/OvHV‑2gB-IN group. As a result, the immunogenicity of the intranasal route for this vaccine candidate cannot be fully assessed in this study, and additional investigations will be required. To further characterize the magnitude of the neutralizing response in groups that showed positive activity, antibody titers were measured using serial dilutions in animals that received the vaccine candidate with or without adjuvant by intramuscular route. At 35 DPI, the average virus‑neutralizing titer in plasma from the rAlHV‑1/OvHV‑2gB‑IM group was 32, whereas the Vac group (rAlHV‑1/OvHV‑2gB plus Emulsigen^®^‑IM) reached an average titer of 64, suggesting that the presence of an adjuvant contributes to a more robust antibody response.

Overall, these results indicate that the absence of an adjuvant led to reduced, but not abolished, humoral responses in a route‑dependent manner. They also suggest that the inclusion of adjuvant can enhance the magnitude of the humoral response following intramuscular immunization.

**Supplementary Figures:**

**Supplementary Figure 1. Cattle OvHV-2 gB-specific IgG response in plasma and nasal secretion following immunization with rAlHV-1/OvHV-2gB.** Calves (n = 4 per group) were immunized by either intramuscular or intranasal routes at 0, 14 and 28 days. Plasma and nasal secretions (NS) were tested at 1:100 and 1:40 dilutions, respectively, and results are shown as a mean Sample/Background OD_450_ ratio of OvHV-2 gB-specific IgG obtained by ELISA, with error bars showing standard deviation. Data for each group and sample type was compared using One-way ANOVA (with Geisser-Greenhouse correction) followed by Dunnett's test for comparisons to 0 days post-prime immunization (DPI). Significant differences between groups are represented by asterisks: *, p < 0.05 and **, p < 0.01.

**Supplementary Figure 2. Overall antibody neutralizing activity following immunization.** rBoHV-4/OvHV-2gB was used to assess neutralizing antibody activity in plasma **(A)** and nasal secretion **(B)** from calves (n = 4 per group) immunized with rAlHV-1/OvHV-2gB without adjuvant either intramuscularly (IM) or intranasally (IN). Both plasma and nasal secretion samples were tested at 1:8. Data from calves immunized intramuscularly with rAlHV-1/OvHV-2gB in presence of adjuvant (Vac) or in the control (Mock) were added as a reference. All samples were obtained at 0 and 35 days post-prime immunization (DPI). Bars represent mean percentage of inhibition, dots indicate individual animals, and error bars are standard deviation. Dotted lines indicate the cutoff (56%) for positive neutralizing activity. Data were compared using t-test and significant differences between groups are represented by asterisks: *, p < 0.05; and ****, p < 0.0001.

**Supplementary Figure 3.** **Experimental design, sampling schedule and testing for the Vac and Mock groups.**

**Supplementary Figure 4. Effect of Emulsigen^®^ on rAlHV-1/OvHV-2gB viability.** rAlHV-1/OvHV-2gB (10^4.67^ TCID_50_ / mL) was incubated with or without 20 % Emulsigen^®^ at 20 °C for 1 h. Following incubation, virus preparations were titrated by limiting dilution assay. Virus titers from three independent experiments are presented as group median with error bars indicating 95% confidence interval. Mann-Whittney test comparing presence and absence of Emulsigen^®^ resulted in no statistical difference (ns) between the groups (p > 0.05).

**Supplementary Tables:**

**Supplementary Table 1.** Antibodies used for cellular immunophenotyping

| **Specificity** | **Clone** | **Source** | **1^ary^ Ab Label** | **1^ary^ Conc (**µg/mL) | **2^ary^ Ab Label** | **2^ary^ Ab Dilution** |
| --- | --- | --- | --- | --- | --- | --- |
| CD3 | MM1A | MAC-WSU | Unlabeled | 3.75 | FITC | 1/100 |
| CD4 | CC8 | Bio-Rad | PE | 2.50 | na | - |
|  | CC8 | Bio-Rad | FITC | 5.00 | na | - |
| CD8 | CC63 | Bio-Rad | PB | 2.50 | na | - |
|  | CC63 | Bio-Rad | FITC | 5.00 | na | - |
| CD14 | CAM36A | MAC-WSU | Unlabeled | 3.75 | FITC | 1/100 |
| CD16 | KD1 | Bio-Rad | Unlabeled | 3.75 | DL650 | 1/100 |
| CD335 | EC1.1 | MAC-WSU | Unlabeled | 3.75 | FITC | 1/100 |
| γδ-TCR | GB21A | MAC-WSU | Unlabeled | 3.75 | DL650 | 1/100 |
| IgM | BAQ155A | MAC-WSU | Unlabeled | 3.75 | FITC | 1/500 |

na: not applicable; 1^ary^/2^ary^: primary/secondary; Ab: antibody; MAC-WSU: Monoclonal Antibody Center, College of Veterinary Medicine, Washington State University, Pullman WA, USA. Bio-Rad Laboratories Inc, Hercules, USA

| **Supplementary Table 2.** Body temperature (^o^C) in vaccinated (V1-V4) and mock (M1-M4) calves throughout the experiment | | | | | | | | | | | | | | | |
| --- | --- | --- | --- | --- | --- | --- | --- | --- | --- | --- | --- | --- | --- | --- | --- |
|  |  |  |  |  |  |  |  |  |  |  |  |  |  |  |  |
| **DPI** | **Vaccinated** | | | | | | |  | **Mock** | | | | | | |
|  | **V1** | **V2** | **V3** | **V4** |  | **Mean** | **SD** |  | **M1** | **M2** | **M3** | **M4** |  | **Mean** | **SD** |
| -1 | 37.8 | 37.9 | 38.5 | 38.5 |  | 38.2 | 0.4 |  | 38.2 | 37.9 | 37.8 | 38.2 |  | 38.0 | 0.2 |
| **0** | 38.3 | 38.8 | 38.7 | 38.8 |  | 38.7 | 0.2 |  | 38.6 | 38.6 | 38.4 | 39.1 |  | 38.7 | 0.3 |
| 1 | 38.4 | 38.4 | 38.9 | 39.2 |  | 38.7 | 0.4 |  | 39.1 | 39.4 | 38.1 | 38.8 |  | 38.9 | 0.6 |
| 2 | 38.4 | 38.9 | 39.8 | 39.3 |  | 39.1 | 0.6 |  | 39.4 | 39.3 | 38.3 | 38.4 |  | 38.9 | 0.6 |
| 3 | 38.9 | 38.1 | 39.7 | 39.3 |  | 39.0 | 0.7 |  | 39.1 | 39.0 | 38.4 | 38.4 |  | 38.7 | 0.4 |
| 4 | 38.1 | 38.1 | 38.5 | 38.8 |  | 38.4 | 0.3 |  | 38.3 | 38.5 | 37.8 | 37.8 |  | 38.1 | 0.4 |
| 5 | 38.4 | 38.8 | 39.0 | 38.9 |  | 38.8 | 0.3 |  | 38.5 | 38.0 | 37.9 | 37.9 |  | 38.1 | 0.3 |
| 6 | 37.9 | 38.1 | 37.8 | 39.0 |  | 38.2 | 0.5 |  | 38.5 | 38.0 | 37.9 | 37.9 |  | 38.1 | 0.3 |
| 7 | 40.2 | 40.1 | 39.0 | 38.6 |  | 39.5 | 0.8 |  | 37.9 | 38.4 | 38.1 | 38.2 |  | 38.2 | 0.2 |
| 8 | 38.8 | 38.7 | 39.5 | 40.3 |  | 39.3 | 0.7 |  | 38.1 | 38.1 | 38.2 | 38.2 |  | 38.2 | 0.1 |
| 9 | 38.5 | 38.7 | 38.3 | 38.7 |  | 38.6 | 0.2 |  | 38.3 | 38.1 | 38.7 | 39.0 |  | 38.5 | 0.4 |
| 10 | 38.2 | 38.2 | 38.2 | 38.6 |  | 38.3 | 0.2 |  | 37.9 | 38.4 | 37.8 | 37.9 |  | 38.0 | 0.3 |
| 11 | 38.4 | 38.3 | 39.3 | 38.5 |  | 38.6 | 0.5 |  | 37.3 | 37.8 | 38.3 | 37.8 |  | 37.8 | 0.4 |
| 12 | 38.0 | 37.9 | 39.1 | 38.5 |  | 38.4 | 0.6 |  | 38.0 | 38.0 | 38.1 | 37.8 |  | 38.0 | 0.1 |
| 13 | 37.3 | 37.7 | 38.2 | 38.3 |  | 37.9 | 0.5 |  | 38.0 | 38.2 | 37.6 | 38.3 |  | 38.0 | 0.3 |
| **14** | 37.7 | 37.8 | 38.3 | 39.0 |  | 38.2 | 0.6 |  | 38.1 | 38.1 | 37.7 | 38.3 |  | 38.1 | 0.3 |
| 15 | 39.2 | 39.4 | 39.7 | 39.1 |  | 39.4 | 0.3 |  | 38.8 | 38.9 | 38.3 | 38.9 |  | 38.7 | 0.3 |
| 16 | 38.0 | 37.7 | 38.4 | 39.0 |  | 38.3 | 0.6 |  | 38.9 | 37.8 | 37.8 | 38.0 |  | 38.1 | 0.5 |
| 17 | 37.6 | 37.4 | 38.7 | 38.8 |  | 38.1 | 0.7 |  | 37.5 | 37.3 | 37.6 | 37.2 |  | 37.4 | 0.2 |
| 18 | 38.4 | 38.2 | 38.3 | 38.6 |  | 38.4 | 0.2 |  | 38.3 | 38.6 | 37.3 | 37.6 |  | 38.0 | 0.6 |
| 19 | 38.1 | 38.5 | 38.8 | 38.2 |  | 38.4 | 0.3 |  | 38.4 | 37.5 | 37.8 | 37.9 |  | 37.9 | 0.4 |
| 20 | 37.9 | 38.3 | 38.2 | 38.4 |  | 38.2 | 0.2 |  | 38.1 | 37.4 | 37.6 | 37.8 |  | 37.7 | 0.3 |
| 21 | 38.6 | 38.3 | 38.8 | 38.7 |  | 38.6 | 0.2 |  | 38.1 | 37.8 | 38.1 | 38.7 |  | 38.2 | 0.4 |
| 22 | 38.0 | 38.6 | 38.4 | 38.5 |  | 38.4 | 0.3 |  | 37.4 | 37.5 | 37.7 | 37.9 |  | 37.6 | 0.2 |
| 23 | 38.4 | 38.4 | 38.7 | 37.8 |  | 38.3 | 0.4 |  | 38.4 | 37.9 | 37.6 | 38.0 |  | 38.0 | 0.3 |
| 24 | 37.9 | 38.3 | 37.7 | 38.1 |  | 38.0 | 0.3 |  | 38.3 | 37.7 | 37.4 | 37.9 |  | 37.8 | 0.4 |
| 25 | 38.8 | 38.7 | 38.8 | 39.0 |  | 38.8 | 0.1 |  | 38.5 | 38.3 | 38.6 | 37.9 |  | 38.3 | 0.3 |
| 26 | 38.5 | 38.1 | 37.7 | 38.5 |  | 38.2 | 0.4 |  | 38.3 | 37.9 | 38.7 | 37.6 |  | 38.1 | 0.5 |
| 27 | 38.3 | 38.3 | 38.2 | 38.2 |  | 38.3 | 0.1 |  | 37.9 | 37.9 | 37.9 | 38.3 |  | 38.0 | 0.2 |
| **28** | 38.3 | 38.3 | 38.7 | 38.5 |  | 38.5 | 0.2 |  | 38.6 | 38.4 | 38.2 | 38.7 |  | 38.5 | 0.2 |
| 29 | 38.4 | 39.1 | 38.3 | 38.6 |  | 38.6 | 0.4 |  | 38.4 | 37.9 | 37.9 | 38.1 |  | 38.1 | 0.2 |
| 30 | 38.3 | 38.8 | 38.6 | 38.8 |  | 38.6 | 0.2 |  | 38.3 | 38.8 | 37.8 | 37.9 |  | 38.2 | 0.5 |
| 31 | 38.8 | 38.4 | 38.4 | 38.0 |  | 38.4 | 0.3 |  | 38.2 | 38.3 | 38.1 | 38.5 |  | 38.3 | 0.2 |
| 32 | 38.2 | 38.2 | 38.8 | 38.7 |  | 38.5 | 0.3 |  | 38.3 | 37.8 | 38.1 | 38.2 |  | 38.1 | 0.2 |
| 33 | 38.6 | 38.6 | 38.6 | 38.8 |  | 38.7 | 0.1 |  | 38.5 | 38.4 | 37.8 | 38.2 |  | 38.2 | 0.3 |
| 34 | 38.7 | 38.1 | 38.1 | 38.6 |  | 38.4 | 0.3 |  | 38.3 | 37.9 | 38.1 | 38.3 |  | 38.2 | 0.2 |
| 35 | 38.8 | 38.6 | 38.8 | 39.1 |  | 38.8 | 0.2 |  | 38.4 | 38.6 | 38.1 | 38.8 |  | 38.5 | 0.3 |
| 36 | 38.6 | 38.5 | 38.9 | 38.7 |  | 38.7 | 0.2 |  | 38.2 | 38.1 | 38.9 | 38.9 |  | 38.5 | 0.4 |
| 37 | 38.9 | 38.7 | 39.0 | 38.9 |  | 38.9 | 0.1 |  | 38.4 | 38.9 | 38.3 | 38.3 |  | 38.5 | 0.3 |
| 38 | 38.5 | 38.3 | 39.0 | 38.5 |  | 38.6 | 0.3 |  | 38.6 | 38.1 | 38.4 | 38.1 |  | 38.3 | 0.2 |
| 39 | 38.9 | 39.2 | 38.8 | 39.0 |  | 39.0 | 0.2 |  | 38.8 | 38.4 | 38.4 | 38.8 |  | 38.6 | 0.2 |
| 40 | 37.7 | 38.6 | 38.5 | 38.3 |  | 38.3 | 0.4 |  | 38.1 | 37.9 | 38.7 | 38.0 |  | 38.2 | 0.4 |
| 41 | 38.9 | 38.6 | 38.6 | 38.8 |  | 38.7 | 0.2 |  | 38.4 | 38.3 | 38.3 | 38.3 |  | 38.3 | 0.1 |
| 42 | 38.8 | 38.2 | 38.3 | 38.7 |  | 38.5 | 0.3 |  | 38.5 | 38.6 | 38.7 | 39.0 |  | 38.7 | 0.2 |
| 43 | 38.2 | 38.6 | 38.9 | 38.2 |  | 38.5 | 0.3 |  | 38.6 | 38.5 | 38.3 | 38.7 |  | 38.5 | 0.2 |
| 44 | 38.6 | 38.2 | 38.3 | 38.5 |  | 38.4 | 0.2 |  | 38.6 | 38.5 | 38.2 | 38.1 |  | 38.4 | 0.2 |
| 45 | 37.9 | 38.6 | 38.8 | 38.6 |  | 38.5 | 0.4 |  | 38.5 | 38.7 | 38.1 | 38.7 |  | 38.5 | 0.3 |
| 46 | 37.8 | 38.1 | 38.0 | 37.8 |  | 37.9 | 0.2 |  | 38.4 | 37.9 | 37.8 | 38.1 |  | 38.1 | 0.3 |
| 47 | 38.1 | 38.5 | 38.6 | 38.2 |  | 38.4 | 0.2 |  | 38.3 | 37.8 | 38.3 | 38.4 |  | 38.2 | 0.3 |
| 48 | 37.9 | 38.4 | 38.1 | 38.7 |  | 38.3 | 0.4 |  | 38.1 | 38.4 | 38.4 | 38.6 |  | 38.4 | 0.2 |
| 49 | 37.9 | 37.8 | 37.1 | 37.9 |  | 37.7 | 0.4 |  | 38.4 | 36.9 | 37.3 | 37.9 |  | 37.6 | 0.7 |
| 50 | 38.2 | 38.6 | 38.3 | 38.5 |  | 38.4 | 0.2 |  | 38.2 | 38.0 | 37.9 | 38.1 |  | 38.1 | 0.1 |
| 52 | 37.8 | 37.9 | 37.9 | 38.3 |  | 38.0 | 0.2 |  | 38.2 | 38.1 | 38.2 | 38.6 |  | 38.3 | 0.2 |
| 55 | 37.5 | 38.0 | 37.9 | 38.5 |  | 38.0 | 0.4 |  | 37.6 | 37.6 | 37.9 | 37.6 |  | 37.7 | 0.2 |
| 57 | 38.3 | 38.6 | 38.8 | 38.5 |  | 38.6 | 0.2 |  | 38.3 | 38.4 | 38.3 | 38.1 |  | 38.3 | 0.1 |
| 59 | 37.9 | 38.5 | 37.8 | 38.1 |  | 38.1 | 0.3 |  | 37.9 | 37.9 | 38.0 | 38.6 |  | 38.1 | 0.3 |
| 62 | 37.3 | 38.1 | 38.3 | 38.0 |  | 37.9 | 0.4 |  | 37.9 | 37.6 | 37.9 | 38.4 |  | 38.0 | 0.3 |
| 64 | 38.9 | 38.3 | 37.9 | 38.8 |  | 38.5 | 0.5 |  | 38.7 | 38.8 | 38.9 | 38.9 |  | 38.8 | 0.1 |
| 66 | 38.1 | 38.5 | 38.6 | 37.8 |  | 38.3 | 0.4 |  | 38.2 | 38.0 | 37.9 | 38.6 |  | 38.2 | 0.3 |
| 69 | 37.1 | 37.9 | 37.7 | 38.2 |  | 37.7 | 0.5 |  | 38.4 | 38.0 | 37.9 | 38.3 |  | 38.2 | 0.2 |
| 70 | 38.5 | 38.9 | 38.2 | 38.8 |  | 38.6 | 0.3 |  | 37.9 | 38.0 | 38.1 | 38.4 |  | 38.1 | 0.2 |
| 71 | 38.5 | 38.9 | 38.6 | 38.8 |  | 38.7 | 0.2 |  | 38.2 | 37.8 | 37.8 | 38.6 |  | 38.1 | 0.4 |
| 76 | 37.9 | 38.1 | 38.7 | 38.2 |  | 38.2 | 0.3 |  | 37.9 | 38.4 | 37.8 | 38.7 |  | 38.2 | 0.4 |
| 84 | 36.8 | 37.9 | 37.8 | 38.0 |  | 37.6 | 0.6 |  | 37.3 | 37.0 | 37.9 | 38.0 |  | 37.6 | 0.5 |

Immunization days indicated in bold and fever (≥39.4^o^C) in red; DPI: days post-prime immunization; SD: standard deviation

**Supplementary Table 3.** Cytokines and chemokines secreted by PBMCs collected from vaccinated (V1-V4) and mock (M1-M4) calves at 24 hours post-last immunization (29 days post prime-immunization) and stimulated in vitro with rAlHV-1/OvHV-2gB for 72 hours

|  | **Net concentration (pg/mL)*** | | | | | | | | | |
| --- | --- | --- | --- | --- | --- | --- | --- | --- | --- | --- |
|  | **V1** | **V2** | **V3** | **V4** |  | **M1** | **M2** | **M3** | **M4** | |
| IL-1𝛼 | 97 | 112 | 82 | 37 |  | 1 | 3 | 1 | 1 |  |
| IL-1β | 223 | 539 | 414 | 226 |  | 2 | 15 | 3 | -5 |  |
| TNF-𝛼 | 385 | 1612 | 633 | 378 |  | 0 | 0 | 0 | 0 |  |
| IFN-γ | 1006 | 3547 | 5396 | 5332 |  | 0 | 0 | 0 | 0 |  |
| IL-4 | 4 | 23 | 10 | 23 |  | 0 | 0 | 2 | 0 |  |
| IL-10 | 57 | 42 | 46 | 69 |  | -8 | 4 | -2 | -3 |  |
| IL-17A | 258 | 332 | 208 | 170 |  | 1 | 2 | 0 | 0 |  |
| VEGF-A | -85 | -123 | -24 | -80 |  | 54 | 56 | 116 | 78 |  |
| CCL3 | 46014 | 45820 | 55524 | 30686 |  | -2 | 7 | 3 | 0 |  |
| CCL4 | 11685 | 12207 | 11843 | 12699 |  | -112 | 588 | 88 | 469 |  |
| CXCL8 | 44165 | 23925 | 27760 | 29488 |  | 306 | 1195 | 509 | 405 |  |
| CXCL10 | 23457 | 3097 | 3253 | 3506 |  | -50 | 142 | 93 | -7 |  |
| IL-6 | 708 | 653 | 378 | 427 |  | 267 | 461 | 341 | 320 |  |
| IL-36RA | -8 | -17 | -7 | 2 |  | -15 | 15 | -12 | -17 |  |
| CCL2 | 2899 | 1957 | 1586 | 1919 |  | -412 | 2310 | 365 | 187 | |

* Cytokine and chemokine concentrations in the supernatants were quantified using a MILLIPLEX^®^ multiplex assay. Net concentrations were determined by subtracting the concentrations measured in matched non-stimulated cells from the corresponding concentrations measured in virus-stimulated cells.
